# High Levels of IL-1β, TNF-α and MIP-1α One Month after the Onset of the Acute SARS-CoV-2 Infection, Predictors of Post COVID-19 in Hospitalized Patients

**DOI:** 10.3390/microorganisms11102396

**Published:** 2023-09-26

**Authors:** Jacobo Alonso-Domínguez, María Gallego-Rodríguez, Inés Martínez-Barros, Beatriz Calderón-Cruz, Virginia Leiro-Fernández, Alexandre Pérez-González, Eva Poveda

**Affiliations:** 1 Virology and Pathogenesis, Galicia Sur Health Research Institute (IIS Galicia Sur), SERGAS-UVIGO, 36312 Vigo, Spain; jacobo.alonso@iisgaliciasur.es (J.A.-D.); maria.gallego@iisgaliciasur.es (M.G.-R.); ines.martinez@iisgaliciasur.es (I.M.-B.); alexandre.perez@iisgaliciasur.es (A.P.-G.); 2 Statistics and Methodology Unit, Galicia Sur Health Research Institute (IIS Galicia Sur), SERGAS-UVIGO, 36312 Vigo, Spain; beatriz.calderon@iisgaliciasur.es; 3 Pneumology Department, Complexo Hospitalario Universitario de Vigo (CHUVI), Sergas, 36312 Vigo, Spain; virginia.leiro.fernandez@sergas.es; 4 NeumoVigo I+i Research Group, Galicia Sur Health Research Institute (IIS Galicia Sur), SERGAS-UVIGO, 36312 Vigo, Spain

**Keywords:** post COVID-19, cytokines, symptomatology

## Abstract

The pandemic caused by SARS-CoV-2 infection has left behind a new symptomatology called post COVID-19, or “long COVID”. The pathophysiological mechanisms still remain controversial; however, a link between persistent inflammation and these sequelae has been suggested. Herein, we longitudinally assessed up- and downstream molecules of the NLRP3 inflammasome’s pathway in three study groups: healthy donors (HC, n = 14) and donors with a confirmed SARS-CoV-2 infection who had been hospitalized, the latter divided into post COVID-19 (PC, n = 27) and non-post COVID-19 patients (nPC, n = 27) based on the presence or absence of symptomatology at month 6, respectively. Plasma cytokines (IL-1β, IL-3, IL-6, IL-8, IL-18, IP-10, MIG, TNF-α, IFN-γ, MIP-1α and MIP-1β) and total peroxide (TPX) levels were quantified at baseline and at months 1 and 6 after the onset of the infection. Baseline values were the highest for both TPX and cytokines that progressively decreased thereafter the acute infection. IL-1β, MIP-1α and TNF-α at month 1 were the only cytokines that showed a significant difference between nPC and PC. These findings suggest that a persistent inflammatory state one month after the onset of SARS-CoV-2 infection related to specific cytokines (IL-1β, MIP-1α, and TNF-α) might guide to predicting post COVID-19 symptomatology.

## 1. Introduction

Coronavirus disease 2019 (COVID-19), caused by severe acute respiratory syndrome coronavirus 2 (SARS-CoV-2), rapidly spread around the world after its outbreak in December 2019, leading to the declaration of a pandemic in early March 2020 by the World Health Organization (WHO) [1]. Since then, around 769 million cases of SARS-CoV-2/COVID-19 infection have been documented.

Although COVID-19 is no longer defined as a Public Health Emergency of International Concern (PHEIC), it continues to take a significant toll on health worldwide. With the pandemic now in its fourth year, it seems clear that the virus is likely to remain with us in the long term. Moreover, there is still a real risk of new variants emerging that could be more transmissible and/or even more severe, which necessitates a continuing need for surveillance and the generation of comprehensive scientific knowledge [2]. COVID-19 could range from asymptomatic cases to mild or severe disease and progress to acute respiratory distress syndrome (ARDS), multiorgan failure and death, especially in older adults and/or those with comorbidities [3]. Most patients will recover spontaneously or after acute-phase management; however, there is a subset of patients who develop a wide range of persistent symptoms [4]. The WHO defined post COVID-19 as a “condition that occurs in individuals with a history of probable or confirmed SARS-CoV-2 infection, usually 3 months from the onset of the infection characterized by the continuation or development of new symptoms and that last for at least 2 months and cannot be explained by an alternative diagnosis” [5]. This includes a range of neurological, musculoskeletal, respiratory and cardiovascular symptoms [6], which may persist for up to, if not more than, 24 months after the infection [7]. Several pathophysiologic mechanisms are being reported in patients with persistent symptoms, including tissue persistence of viral antigen, alterations in the gut microbiome, microvascular dysfunction, generation of autoantibodies against immune proteins such as cytokines, complement and cell surface proteins, and an underlying inflammation [8]. Taking into account all these data, the clinical picture of post COVID-19 should not be attributed to a single pathophysiological mechanism, but rather, this condition is probably described by several interlinked mechanisms.

A relevant area of the research effort is focused on elucidating an inflammatory signature [9]. The proinflammatory environment that surrounds the acute COVID-19 pathology has been characterized by an exacerbated immune response that leads to the release of several cytokines and the generation of higher levels of reactive oxygen species (ROS) [10]. Moreover, in this exacerbated response, NLRP3 is the most-studied inflammasome and the first known to be activated during SARS-CoV-2 [11]. Greater levels of inflammatory molecules in post COVID-19 individuals might be indicative of an ongoing inflammation, which would suggest a possible link between persistent inflammation and post COVID-19 symptoms; however, the available literature related to this topic presents controversial results [12], probably due to the heterogeneity of the methodology used (i.e., including different panels of cytokines and commercial assays for their quantification) and the clinical sampling times evaluated. So far, although there are studies that have found a relationship between some molecules in the post COVID-19 condition [13,14], no predictive biomarker robustly associated with post COVID-19 symptomatology has been identified [15].

Current studies report that approximately 10 to 20% of individuals diagnosed with SARS-CoV-2 infection could potentially exhibit symptoms classifiable as long COVID. While the precise figures pertaining to individuals grappling with this condition remain indeterminate, it is hypothesized that over 17 million individuals within the WHO European Region might have encountered it over the initial biennium of the pandemic (2020/21). It is, therefore, a matter of concern that requires a special compromise to elucidate and face the mechanisms underlying post COVID-19 symptomatology [5].

Thus, the aim of this study was to evaluate whether persistent inflammation after the acute infection is present in patients with post COVID-19 symptoms in an attempt to explore biomarkers in plasma that could be related to the course of the disease and patient’s recovery.

## 2. Materials and Methods

### 2.1. Patients’ Selection and Experimental Design

A prospective longitudinal study was performed by selecting individuals from the Galicia Sur Health Research Institute COVID-19 Cohort (COHVID-GS) (https://www.iisgaliciasur.es/apoyo-a-la-investigacion/cohorte-covid19 [accessed on 30 August 2023]). This cohort includes patients with SARS-CoV-2 confirmed by PCR in nasopharyngeal exudate under clinical follow-up in the Vigo Healthcare Area with epidemiological/clinical data and with a repository of biological samples (i.e., nasopharyngeal swabs, whole blood, plasma, serum and peripheral blood mononuclear cells—PBMCs) stored at the Galicia Sur Health Research Institute Biobank. The epidemiological/clinical information was collected in a case report form (CRF) specifically designed for the COHVID-GS. The cohort also includes a control group of uninfected individuals (PCR, anti-IgA, anti-IgM, and anti-IgG SARS-CoV-2 negative).

Three groups were defined: healthy donors without SARS-CoV-2 infection and donors with a confirmed SARS-CoV-2 diagnosis who had been hospitalized, further divided into post COVID (PC) and non-post COVID (nPC) patients. All the patients included had plasma samples at baseline (maximum 10 days after the confirmation of SARS-CoV-2) and months 1 and 6 after the acute infection. PC patients were distinguished from nPC based on the presence or absence of symptoms at month six, respectively, which was determined by an infectious diseases physician in a specific clinical consultation for the follow-up of COVID-19 patients and determination of whether they meet post COVID symptomatology. Individuals between groups and healthy donors were matched by sex/age. Epidemiological and clinical characteristics were collected (i.e., symptoms on admission and month six, comorbidities or severity).

### 2.2. Plasma Cytokines Quantification

A multiplex bead-based immunoassay (MILLIPLEX^®^ Human Cytokine/Chemokine Magnetic Bead Panel, Merck KGaA, Darmstadt, Germany) was used to quantify the levels of the following plasma cytokines: interleukin 1 beta (IL-1β), interleukin-3 (IL-3), interleukin-6 (IL-6), interleukin-8 (IL-8), interleukin-18 (IL-18), interferon-gamma induced protein 10 (IP-10), monokine induced by gamma interferon (MIG), tumor necrosis factor-alpha (TNF-α), interferon-gamma (IFN-γ) and macrophage inflammatory proteins 1 alpha (MIP-1α) and 1 beta (MIP-1ꞵ). The main functions of these molecules are described in Table S1. The assays followed the manufacturer’s instructions and chosen factors have been analyzed on the MAGPIX^®^ platform (Luminex Corporation, Austin, TX, USA).

### 2.3. Reactive Oxygen Species (ROS) Quantification

ROS values were analyzed by measuring total peroxides. According to the user’s manual, the total peroxide concentration (TPX) was measured in plasma samples using OxyStat colorimetric assay kit (Biomedica, Wien, Austria). This technique allows the assessment of the color change produced by the reaction of total peroxide in the sample with peroxidase performing two measures. Absorption was detected using a FLUOstar Omega (BMG LABTECH, Ortenberg, Germany) at 450 nm and the OD values were proportional to TPX.

### 2.4. Statistical Analysis

The descriptive analyses were reported as frequencies and percentages for categorical variables, and medians and interquartile ranges (IQRs) for continuous variables. Since the study groups consisted of less than 30 individuals, non-parametric tests were applied for analysis. Fisher’s exact test and chi square test were performed to evaluate the differences in clinical values between groups. To assess the changes in the cytokines and ROS dynamics of each group, the Friedman test was used. To compare the cytokine and ROS concentration between HC, nPC, and PC a Kruskal–Wallis test was performed, followed by Dunn’s test for pairwise multiple comparisons when the Kruskal–Wallis test was significant. Additionally, we used the Mann–Whitney U test to compare cytokine concentrations between nPC and PC. Furthermore, we performed a ROC curve analysis to estimate the optimal cutoff for IL-1ꞵ, TNF-α and MIP-1α concentrations that could predict post COVID-19 symptomatology.

Statistical analyses were performed with SPSS (v26 IBM Corp, Armonk, NY, USA) and GraphPad Prism (v8.0.1, GraphPad Software, San Diego, CA, USA). *p* values < 0.05 were considered statistically significant.

## 3. Results

### 3.1. Demographic and Clinical Characteristics of the Study Population

A total of 68 individuals were included: 27 nPC, 27 PC and 14 healthy controls. The most relevant clinical and demographic characteristics related to our cohort are shown in Table 1. The median age of males and females was 58 and 62 years old, respectively [IQR: 52.5–63.5 vs. 21–68.5]. A total of 20 patients (37.04%) met the obesity criteria (BMI ≥ 30). The median hospitalization time was 7 days [4–9.75], and 20.37% were admitted to an intensive care unit (ICU), and eight of them required invasive mechanical ventilation. We performed Fisher’s exact test to check if there were differences between the characteristics of the study population corresponding to the PC and nPC groups; however, no differences were observed between these two groups. Table 2 shows the most prevalent COVID-19 symptoms in the study population.

### 3.2. Post COVID-19 Symptomatology

For those 27 individuals who met the criteria of PC, Table 3 summarizes the categories of post COVID-19 symptomatology presented in the study population. 16/27 (59.3%) showed thoracic symptoms, with dyspnea (the first grade on the MCR scale) being the most frequent one, 7/27 (25.9%) showed nervous system symptoms and 7/27 had musculoskeletal symptoms. Specifically, asthenia (37.0%) and hair loss (14.8%) were the predominant general symptoms among individuals.

### 3.3. Non-Post and Post COVID-19 Cytokine Dynamics

**Figure 1:**
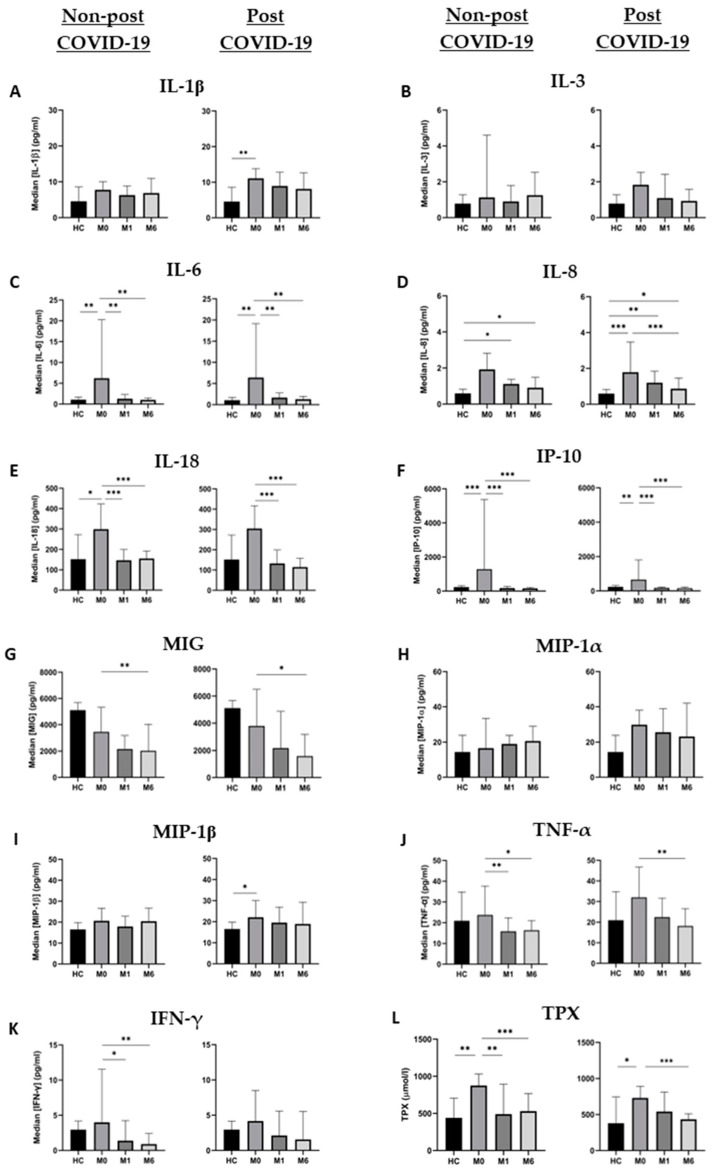
Cytokine and TPX dynamics in nPC and PC during the study period. (**A**) IL-1ꞵ, (**B)** IL-3, (**C**) IL-6, (**D**) IL-8, (**E**) IL-18, (**F**) IP-10, (**G**) MIG, (**H**) MIP-1α, (**I**) MIP-1ꞵ, (**J**) TNF-α, (**K**) IFN-γ and (**L**) TPX levels are expressed as median ± IQR in the three tested time points. Changes between different time points of each group were analyzed by the Friedman test, and differences between groups at each time point were assessed using the Kruskal–Wallis test. * = *p* value < 0.05; ** = *p* value < 0.01; *** = *p* value < 0.001.

Plasma cytokine measurements were performed in 54 patients at the three time points and in 14 healthy controls. Figure 1 shows the dynamics of the molecules assessed and Tables S2 and S3 list the number of samples, median, IQR and *p* values obtained from the molecules analyzed.

All molecules assessed were at the highest points of their dynamics during acute infection. More specifically, baseline IL-6 and IP-10 levels of both groups, IL-18 levels in nPC and IL-1ꞵ and IL-8 in PC were significantly higher than HC values (Figure 1). Once the acute infection was resolved, plasma levels of cytokines showed a progressive decrease during the follow-up, most of them reaching the levels of HC at month 6, with the most pronounced decreases in IL-6 and IP-10.

**Figure 2:**
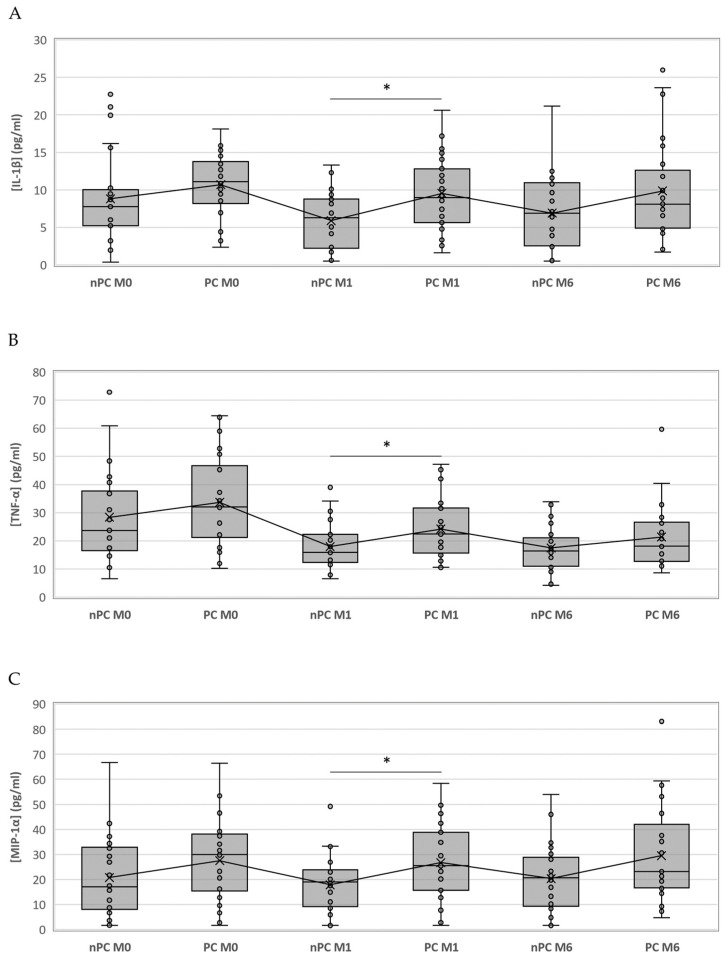
Boxplot representing the differences of IL-1ꞵ, TNF-α and MIP-1α among nPC and PC. IL-1ꞵ (**A**), TNF-α (**B**) and MIP-1α (**C**) concentrations in PC and nPC. Differences between groups were assessed using the Kruskal–Wallis test and the Mann–Whitney U test when appropriate. * = *p* value < 0.05.

We compared the same time points for each cytokine between the study groups; however, only IL-1ꞵ showed statistically significant differences at month 1 (*p* = 0.024), where the PC group had higher values than nPC. Nevertheless, since the significances obtained in the Kruskal–Wallis test for MIP-1α and TNF-α at month 1 were close to *p* < 0.05 (0.059 and 0.056, respectively), we compared medians between nPC and PC with the Mann–Whitney U test. We were able to identify statistically significant differences for TNF-α (*p* = 0.026) and MIP-1α (*p* = 0.020) at month 1. Although the only significant difference appeared at month 1, cytokine concentrations for PC were above nPC thereafter (Figure 2).

### 3.4. Non-Post and Post COVID-19 Total Peroxides Dynamics

Total peroxide (TPX) concentration was also measured in the same samples and timepoints. Figure 1 shows the dynamic of the median concentrations of TPX. Levels at baseline were significantly higher for nPC and PC individuals compared to HC, and these levels significantly decreased during the follow-up for both groups. However, we have observed that the decrease in TPX levels in PC patients occurs more slowly than in nPC. Nevertheless, in both cases values at month 6 reach almost the same levels as those of healthy controls. Moreover, no significant differences were observed between PC and nPC at any of the three assessed time points.

### 3.5. Predictive Model for Post COVID-19

**Figure 3:**
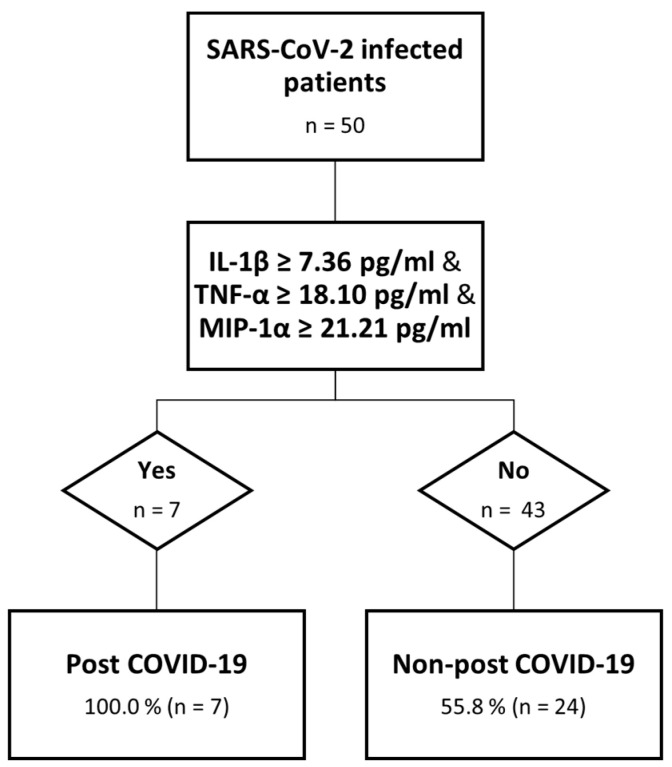
Decision tree analysis. Predictive model of post COVID-19.

We were able to identify, for those cytokines with higher values among PC than nPC at month 1, the best cut-off point using a ROC analysis for IL-1ꞵ (7.36 μg/mL and AUC: 0.709), MIP-1α (21.20 μg/mL and AUC: 0.691) and TNF-α (18.1 μg/mL and AUC: 0.682) above which the risk of having post COVID-19 symptoms at month 6 was significantly higher. Based on these data, and to evaluate the risk of developing post COVID-19 symptomatology, we created a “Risk” variable to divide those who surpassed all the established cut-offs from those who did not. We performed a χ^2^ test to assess the relation between “Risk” and post COVID-19 symptomatology, identifying seven patients who had surpassed these values, all of them belonging to the PC group. For this relationship, a *p* = 0.010 was obtained. We explored other potential combinations: IL-1ꞵ/TNF-α (*p* = 0.003), IL-1ꞵ/MIP-1α (*p* = 0.003), and TNF-α/MIP-1α (*p* = 0.103), where the first two combinations correctly classify 90.9 and 80.0%, respectively, of the post COVID-19 cases (Figure 3).

## 4. Discussion

As the COVID-19 pandemic persists and new cases of post COVID-19 are being reported, there is a concern about the health consequences in the medium- and long-term for people with persistent COVID-19 symptomatology, some of them for more than two years. Moreover, neither a biomarker for early diagnosis and clinical follow-up of post COVID nor a biological target to prevent, relieve or cure post COVID condition have yet been identified. Therefore, the aim of this study was to identify potential biomarkers for post COVID-19 symptoms that can add knowledge for a comprehensive approach to the underlying mechanisms. We report the highest levels for both TPX and cytokines during the acute phase of the infection that progressively decreased thereafter. We were able to identify IL-1β, MIP-1α and TNF-α at month 1 as the only cytokines that show a significant difference between nPC and PC. which allowed us to define an accurate model to predict post COVID-19 symptomatology early, as long as the levels of these three cytokines exceed their respective established cut-offs. However, the specificity of the model is 55.8%, which indicates that patients with lower levels of theses cytokines do not rule out the possibility of suffering this symptomatology.

Several studies have explored the link existing between SARS-CoV-2 infection and the inflammatory processes, named the “cytokine storm” [16], considered one of the most important causative events during COVID-19 disease. It has been well described how levels of specific cytokines involved in this phenomenon are increased [17,18], leading to the development of a deregulated response and proinflammatory environment, which is related to the degree of severity and the outcome of COVID-19. Overall, elevated serum levels of some cytokines have been well associated with more severe disease [19]. In our study, significantly higher levels of IL-6, MIP-1β, IP-10, IL-8, IL-1β, IL-18 and TPX were observed during the onset of the acute phase of SARS-CoV-2 infection compared to healthy controls. This pattern of cytokine dynamics has also been observed during the acute phases of other infections of the same genus, such as SARS and MERS [20,21]. Similarly to this study, during the course of the aforementioned infections, after the peaks of cytokines levels during the acute phase a progressive decrease is observed thereafter. For both infections, the cytokines levels were also associated with the severity and outcome of the disease.

It has been attempted to define the inflammatory profile that might define post COVID-19 symptomatology. Nevertheless, no plasma biomarker able to predict post COVID-19 early on has been found yet. Among all the molecules we assessed, IL-1β, IL-18 and TPX are the main components of the NLRP3 inflammasome pathway, which is able to recognize RNA viruses [22] and induce the release of these molecules, leading to the cytokine storm. Our results have allowed the design of a predictive model for post COVID-19 based on the levels of three cytokines at month 1 (IL-1β, TNF-α, and MIP-1α). For our cohort, all the patients whose values of these three molecules surpassed the cut-off established presented post COVID-19 symptomatology at month 6. Thus, elevated levels of cytokines one month after the infection could be a preliminary explanation for post COVID-19 condition. IL-1β, TNF-α and MIP-1α play different roles in inflammation [23], so this hypothesis might be consistent with the persistent symptomatology.

IL-1β is a potent proinflammatory cytokine that is crucial for host-defense responses to infection. It also exacerbates damage during chronic disease and acute tissue injury. There is plenty of literature linking it to COVID-19. In fact, several studies showed how blocking the release of this cytokine might improve patients’ condition [24]. Elevated ROS levels are not necessary to reach these values of IL-1β at month 1, since viral antigens could also start the inflammasome pathway, explaining the maintenance of IL-1β levels [25]. Bertoni et al. demonstrated that the SARS-CoV-2 protein ORF3a activates the NLRP3 inflammasome in vitro [26]. Moreover, it has been demonstrated that SARS-CoV-2 nucleocapsid protein (N) can directly interact with NLRP3 to promote the assembly and activation of the inflammasome, thus leading to the production of a large number of inflammatory molecules in mice [27].

The levels of TNF-α at the three time points in PC are higher than in nPC. This complies with the existing literature, where higher TNF-α values have also been demonstrated in individuals with post COVID-19 symptomatology [28]. This molecule is also implicated in the cytokine storm, which is consistent with our cytokine hypothesis, where the dysregulation of the inflammatory response through a positive feedback loop leads to even more cytokine production and inflammation.

Higher values of MIP-1α in the PC group, a cytokine involved in the recruitment and activation of immune cells (particularly monocytes and macrophages) to sites of infection, are in line with existing literature both in baseline levels during acute COVID-19 and six months after infection [29].

These three cytokines can together contribute to the persistent immune response, with IL-1β acting as the central axis, being part of the two models that we tested. TNF-α and IL-1β have overlapping pathways, similar to NF-κB, and can act synergistically to enhance the inflammatory response. They can induce the expression of each other, creating a positive feedback loop that amplifies their effects. In addition, it has been described that the production of MIP-1α can be enhanced by IL-1β through the activation of NF-κB.

Several pathophysiologic mechanisms have been suggested to explain persistent symptoms after SARS-CoV-2 infection, including tissue persistence of viral antigen [30], alterations in the gut microbiome [31] and generation of autoantibodies against immune proteins such as cytokines and an underlying inflammation. Therefore, the clinical picture of post COVID-19 might be described by several intertwined mechanisms.

We previously identified female sex or advanced age as risk factors for post COVID-19 symptomatology [32]; therefore, our study population was matched to eliminate these biases and to be able to have more clarity on the physiological factors.

Some limitations should be considered. The small number of the study population for each group may have not been able to achieve enough statistical power to observe significant differences. However, we could identify a specific cytokine profile clearly related to post COVID-19 among this well-defined cohort. In addition, since the PC group is formed by hospitalized COVID-19 survivors, the data obtained could only be extrapolated to this population. Lastly, the collection of symptoms (i.e., headache, anosmia) could be a bias in the study, since patients who were interviewed with a questionnaire could have had a subjective view of their own conditions that does not accurately reflect reality. Although we are aware of the limitations of this study, these do not prevent us from reaching reliable conclusions.

In summary, we were able to establish an accurate model to early predict post COVID-19 symptomatology based on IL-1β, TNF-α and MIP-1α levels one month after the onset of the acute SARS-CoV-2 infection. Persistent inflammation shortly after the onset of the acute infection through the NLRP3 inflammasome pathway might explain, at least in part, the post COVID-19 phenomenon. Our results suggest the involvement of this molecule during the acute phase of SARS-CoV-2 infection by measuring the increase of both the concentration of one of the NLRP3 activators and its products. The validation of these results in other cohorts would serve to confirm this model as an early diagnostic strategy.

## Figures and Tables

**Table 1:** Demographic and Clinical Characteristics of the Study Population.

	Controls	Total	Post COVID	Non-Post COVID	*p*-Value
Patients in follow-up, n (%)	14	54 (100%)	27 (50%)	27 (50%)	
**Demographics**					
Age, in years, median [IQR]	53.5 [47–57]	59.5 [51.25–68]	58 [52–64]	62 [51–68.5]	*p* = 0.342
<55	7 (50%)	18 (33.33%)	10 (37.04%)	8 (29.63%)	
≥55	7 (50%)	36 (66.67%)	17 (62.96%)	19 (70.37%)	
Male sex, n (%)	8 (57.14)	22 (40.74%)	11 (40.74%)	11 (40.74%)	*p* = 1.000
**Comorbidities**					
Obesity, n (%)	2 (14.29%)	20 (37.04%)	12 (44.44%)	8 (29.63%)	*p* = 0.398
Hypertension, n (%)	1 (7.14%)	17 (31.48%)	8 (29.63%)	9 (33.33%)	*p* = 1.000
Chronic obstructive pulmonary disease, n (%)	0 (0%)	4 (7.41%)	3 (11.11%)	1 (3.70%)	*p* = 0.610
Diabetes mellitus, n (%)	0 (0%)	10 (18.52%)	4 (14.81%)	6 (22.22%)	*p* = 0.728
Asthma, n (%)	0 (0%)	4 (7.41%)	4 (14.81%)	0 (0%)	*p* = 0.111
HIV infection, n (%)	0 (0%)	2 (3.70%)	2 (7.4%)	0 (0%)	*p* = 0.491
Chronic kidney disease, n (%)	0 (0%)	1 (1.85%)	1 (3.7%)	0 (0%)	*p* = 1.000
Chronic inflammatory disease	0 (0%)	2 (3.7%)	1 (3.7%)	1 (3.7%)	*p* = 1.000
**Severity**					
Days from symptoms onset to SARS-CoV-2 confirmation, median [IQR]		4 [2–7]	4 [2–9]	5 [3–7]	*p* = 1.000
Days of hospitalization, median [IQR]		7 [4–9.75]	6 [4–15]	8 [5–9]	*p* = 0.610
Admission to ICU, n (%)		11 (20.37%)	6 (22.22%)	5 (18.52%)	*p* = 1.000
Days in ICU, median [IQR]		13 [6–19]	15.5 [12.5–23.75]	8 [5–8]	*p* = 0.113
Invasive mechanical ventilation, n (%)		8 (14.81%)	5 (18.52%)	3 (11.11%)	*p* = 0.704
**Tobacco use**					
Active smoker, n (%)	1 (7.14%)	4 (7.41%)	2 (7.41%)	2 (7.41%)	*p* = 1.000
Former smoker, n (%)	5 (35.71%)	26 (48.15%)	13 (48.15%)	13 (48.15%)	*p* = 1.000

**Table 2:** Symptoms on admission.

	Post COVID	Non-Post COVID	*p*-Value
Patients in follow-up, n	27	27	
**Symptoms on admission**			
Fever, n (%)	21 (77.78%)	20 (74.07%)	*p* = 1.000
Dyspnea, n (%)	17 (62.96%)	19 (70.37%)	*p* = 0.773
Cough, n (%)	17 (62.96%)	17 (62.96%)	*p* = 1.000
Diarrhea, n (%)	9 (33.33%)	9 (33.33%)	*p* = 1.000
Myalgia, n (%)	6 (22.22%)	8 (29.63%)	*p* = 0.757
Anosmia, n (%)	5 (18.52%)	8 (29.63%)	*p* = 0.526
Chest pain, n (%)	8 (29.63%)	2 (7.41%)	*p* = 0.076
ARDS, n (%)	6 (22.22%)	2 (7.41%)	*p* = 0.250

**Table 3:** Symptoms at month 6.

		Post COVID-19
Patients in follow-up, n		27
**Nervous system symptoms, n (%)**		7 (25.93%)
Anosmia, n (%)		1 (3.70%)
Ageusia, n (%)		2 (7.40%)
Headache, n (%)		2 (7.40%)
Migraine, n (%)		1 (3.70%)
Mood disorders, n (%)		1 (3.70%)
Behavioral disorder, n (%)		3 (11.11%)
**Thoracic symptoms, n (%)**		16 (59.25%)
Dyspnea	MRC 1, n (%)	11 (40.74%)
	MRC 2, n (%)	2 (7.40%)
	MRC 3, n (%)	0 (0%)
	MRC 4, n (%)	0 (0%)
Chest pain, n (%)		3 (11.11%)
Cough, n (%)		3 (11.11%)
**Musculoskeletal Symptoms, n (%)**		7 (25.93%)
Myalgias, n (%)		3 (11.11%)
Arthralgias, n (%)		4 (14.81%)
**General symptoms, n (%)**		12 (44.44%)
Asthenia, n (%)		10 (37.03%)
Hair loss, n (%)		4 (14.81%)

## Data Availability

Not applicable.

## Supplementary Materials

The following supporting information can be downloaded at: https://www.mdpi.com/article/10.3390/microorganisms11102396/s1, Table S1: Main functions and properties of the evaluated cytokines; Table S2: Dynamic changes in plasma cytokine levels throughout time in people with post COVID-19 symptomatology; Table S3: Dynamic changes in plasma cytokine levels throughout time in people without post COVID-19 symptomatology.
