# Supplementary material for: High Levels of IL-1β, TNF-α and MIP-1α One Month after the Onset of the Acute SARS-CoV-2 Infection, Predictors of Post COVID-19 in Hospitalized Patients
Source: Microorganisms. 2023 Sep 26;11(10):2396. doi: 10.3390/microorganisms11102396 (PMC10609568; doi:10.3390/microorganisms11102396)
Supplement: Supplementary file 1 [file microorganisms-11-02396-s001.zip › microorganisms-2576617-Supplemental Table S1.pdf]

Supplemental Table S1. Main functions and properties of the evaluated cytokines.

Cytokine	Type	Pro/Anti	Principal function
IL-1β	Interleukin	Pro-inflammatory	The key mediator of the inflammatory response. Essential for host response. It exacerbates damage during chronic disease and acute tissue injury. Promotes leukocyte recruitment to the site of infection. Amplifies the response of T and B lymphocytes. It induces the production of interleukin 6.[1]
IL-3	Interleukin	Adaptative immunity	It is a T cell-derived pluripotent hematopoietic colony-stimulating factor required for the survival and proliferation of primitive hematopoietic progenitor cells. It also regulates the oxidative response in neutrophils. On non-hematopoietic cells, it can cause endothelial cell proliferation and migration, and the proliferation of tumor cell lines.[2]
IL-6	Interleukin	Pro-inflammatory/Anti-inflammatory	It is a pleiotropic cytokine produced in response to tissue damage. It is involved in the production of immunoglobulins, in differentiating B lymphocytes, and in activating and recruiting cytotoxic T lymphocytes and plasma cells. It rescues T cells from entering in apoptosis.[3]
IL-8 (CXCL8)	Chemokine	Pro-inflammatory	IL-8 is a key regulator of the acute inflammatory response, and it recruits and activates monocytes and neutrophils at the site of inflammation. IL-8 also stimulates phagocytosis once they have arrived. IL-8 is also known to be a potent promoter of angiogenesis.[4]
IL-18	Interleukin	Pro-inflammatory	It is a pleiotropic cytokine responsible for regulating both the innate and acquired immune response and plays a fundamental role in autoimmune, inflammatory, and infectious disorders. It accelerates the induction of TH1 cell differentiation. Induces INF-γ and IL-4 production. Possesses chemotactic properties to attract polymorphonuclear cells through induction of IL-8. Increases cytotoxic activity of NK and CD8+ T-cells.[5]
MIG (CXCL9)	Chemokine	Pro-inflammatory	Monokine induced by gamma interferon. It is a chemoattractant for tumor-infiltrating lymphocytes, activated peripheral blood lymphocytes, NK, and Th1 lymphocytes. It is an angiostatin agent. It inhibits neovascularization and has anti-tumor and anti-metastatic effects.[6]
IP-10 (CXCL10)	Chemokine	Pro-inflammatory	Interferon gamma-induced protein-10. It is a chemoattractant for monocytes and T cells. Inhibition of angiogenesis. Promotion of T cell adhesion molecule expression. Induce apoptosis. It inhibits too endothelial healing.[7]
INF-γ	Interferon	Pro-inflammatory	It can inhibit the proliferation of Th2 cells, induce the expansion of Th1 cells, promotes macrophage killing in response to intracellular microbes and parasites, and induce or up-regulates MHC class II expression on immune and nonimmune cells.[8]
TNF-α	Tumor Necrosis Factor	Pro-inflammatory/Anti-inflammatory	It has been identified as a major regulator of inflammatory responses. It can induce systematic inflammation. It exhibits crosstalk with INF- γ to induce the activation of macrophages. It can activate T cells and stimulate NK cells. It contributes to the production of IL-6 and C-reactive proteins.[9]
MIP-1α (CCL3)	Chemokine	Pro-inflammatory	It recruits macrophages, lymphocytes, and eosinophils to the site of inflammation, preferentially attracts activated CD8+ T cells,

			inhibits the hematopoietic cell proliferation, and mediates macrophages migration into wounds to mediate tissue repair.[10]
MIP-1β (CCL4)	Chemokine	Pro-inflammatory	It is a chemoattractant for NK cells, monocytes, T lymphocytes, and dendritic cells at the inflammation site.[10]

1. Lopez-Castejon G, Brough D. Understanding the mechanism of IL-1β secretion. *Cytokine Growth Factor Rev*. 2011;22(4):189-195. doi:10.1016/j.cytogfr.2011.10.001

2. Bénard A, Hansen FJ, Uhle F, et al. Interleukin-3 protects against viral pneumonia in sepsis by enhancing plasmacytoid dendritic cell recruitment into the lungs and T cell priming. *Front Immunol*. 2023;14. doi:10.3389/fimmu.2023.1140630

3. Tanaka T, Narazaki M, Kishimoto T. IL-6 in Inflammation, Immunity, and Disease. *Cold Spring Harb Perspect Biol*. 2014;6(10):a016295-a016295. doi:10.1101/cshperspect.a016295

4. Brennan K, Zheng J. Interleukin 8. In: *XPharm: The Comprehensive Pharmacology Reference*. Elsevier; 2007:1-4. doi:10.1016/B978-008055232-3.61916-6

5. Biet F, Locht C, Kremer L. Immunoregulatory functions of interleukin 18 and its role in defense against bacterial pathogens. *J Mol Med*. 2002;80(3):147-162. doi:10.1007/s00109-001-0307-1

6. Fulkerson PC, Rothenberg ME. CHEMOKINES, CXC | CXCL9 (MIG). In: *Encyclopedia of Respiratory Medicine*. Elsevier; 2006:398-402. doi:10.1016/B0-12-370879-6/00471-3

7. Dufour JH, Dziejman M, Liu MT, Leung JH, Lane TE, Luster AD. IFN-γ-Inducible Protein 10 (IP-10; CXCL10)-Deficient Mice Reveal a Role for IP-10 in Effector T Cell Generation and Trafficking. *The Journal of Immunology*. 2002;168(7):3195-3204. doi:10.4049/jimmunol.168.7.3195

8. Tau G, Rothman P. Biologic functions of the IFN-gamma receptors. *Allergy*. 1999;54(12):1233-1251. doi:10.1034/j.1398-9995.1999.00099.x

9. Atzeni F, Sarzi-Puttini P. Tumor Necrosis Factor. In: *Brenner's Encyclopedia of Genetics*. Elsevier; 2013:229-231. doi:10.1016/B978-0-12-374984-0.01594-1

10. Menten P, Wuyts A, Van Damme J. Macrophage inflammatory protein-1. *Cytokine Growth Factor Rev*. 2002;13(6):455-481. doi:10.1016/S1359-6101(02)00045-X
