# Supplementary material for: High Levels of IL-1β, TNF-α and MIP-1α One Month after the Onset of the Acute SARS-CoV-2 Infection, Predictors of Post COVID-19 in Hospitalized Patients
Source: Microorganisms. 2023 Sep 26;11(10):2396. doi: 10.3390/microorganisms11102396 (PMC10609568; doi:10.3390/microorganisms11102396)
Supplement: Supplementary file 1 [file microorganisms-11-02396-s001.zip › microorganisms-2576617-Supplemental Table S2.pdf]

Supplemental Table S2. Dynamic changes in plasma cytokines levels throughout time in people with post COVID-19 symptomatology.

Post COVID-19

	**INF-γ**			**TNF-α**			**MIG**		
	n	Median [IQR]	*p*-value (versus baseline)	n	Median [IQR]	*p*-value (versus baseline)	n	Median [IQR]	*p*-value (versus baseline)
**M0**	25	4.17 [1.26-8.50]	-	26	32.03 [21.21-46.66]	-	25	3790.83 [2158.73-6495.83]	-
**M1**	25	2.12 [1.18-5.58]	0.447	26	22.45 [15.65-31.69]	0.076	26	2178.92 [1642.76-4866.95]	0.015
**M6**	25	1.56 [1.07-5.53]	0.214	26	18.13 [12.72-26.58]	0.001	25	1584.41 [1387.23-3170.56]	0.117

	**IP-10**			**MIP-1α**			**MIP-1β**		
	n	Median [IQR]	p-value (versus baseline)	n	Median [IQR]	p-value (versus baseline)	n	Median [IQR]	p-value (versus baseline)
**M0**	23	659.23 [267.34-1795.51]	-	27	29.74 [16.24-38.03]	-	25	22.10 [16.68-30.02]	--
**M1**	25	178.10 [120.58-234.33]	0.001	27	25.45 [15.65-38.85]	0.231	27	19.56 [14.96-26.89]	0.214
**M6**	25	158.22 [88.90-225.71]	< 0.001	25	23.18 [16.66-41.99]	1.000	26	18.94 [15.31-29.23]	0.109

	**IL-1β**			**IL-3**			**IL-6**		
	n	Median [IQR]	*p*-value (versus baseline)	n	Median [IQR]	*p*-value (versus baseline)	n	Median [IQR]	*p*-value (versus baseline)
**M0**	26	11.09 [8.19-13.79]	-	27	1.83 [0.55-2.52]	-	24	6.40 [2.16-19.12]	-
**M1**	27	8.94 [5.63-12.81]	0.091	26	1.09 [0.59-2.41]	0.369	25	1.68 [1.13-2.75]	0.008
**M6**	25	8.10 [4.89-12.60]	0.154	21	0.92 [0.64-1.57]	0.428	25	1.28 [1.02-1.97]	0.002

	**IL-8**			**IL-18**			**TPX**		
	n	Median [IQR]	*p*-value (versus baseline)	n	Median [IQR]	*p*-value (versus baseline)	n	Median [IQR]	*p*-value (versus baseline)
**M0**	26	1.78 [1.21-3.47]	-	27	304.34 [118.22-417.28]	-	26	756.42 [575.78-976.46]	-
**M1**	26	1.19 [0.80-1.84]	0.050	23	132.78 [93.04-198.74]	< 0.001	27	541.36 [385.31-813.27]	0.055
**M6**	22	0.86 [0.58-1.46]	<0.001	24	114.88 [62.69-158.49]	< 0.001	27	460.98 [339.91-658.49]	< 0.001
