# Supplementary material for: High Levels of IL-1β, TNF-α and MIP-1α One Month after the Onset of the Acute SARS-CoV-2 Infection, Predictors of Post COVID-19 in Hospitalized Patients
Source: Microorganisms. 2023 Sep 26;11(10):2396. doi: 10.3390/microorganisms11102396 (PMC10609568; doi:10.3390/microorganisms11102396)
Supplement: Supplementary file 1 [file microorganisms-11-02396-s001.zip › microorganisms-2576617-Supplemental Table S3.pdf]

Supplemental Table S3. Dynamic changes in plasma cytokines levels throughout time in people without post COVID-19 symptomatology.

Non-post COVID-19

	**INF-γ**			**TNF-α**			**MIG**		
	n	Median [IQR]	*p*-value (versus baseline)	n	Median [IQR]	*p*-value (versus baseline)	n	Median [IQR]	*p*-value (versus baseline)
**M0**	22	3.99 [2.17-11.53]	-	25	23.73 [16.56-37.70]	-	25	3448.49 [2285.90-5323.5]	-
**M1**	22	1.38 [0.47-4.26]	0.011	25	15.85 [12.35-22.34]	0.008	23	2159.28 [1398.26-3186.29]	0.395
**M6**	22	0.91 [0.64-2.39]	< 0.001	25	16.37 [10.95-21.05]	0.010	27	2027.23 [1461.61-4033.42]	0.002

	**IP-10**			**MIP-1α**			**MIP-1β**		
	n	Median [IQR]	*p*-value (versus baseline)	n	Median [IQR]	p-value (versus baseline)	n	Median [IQR]	p-value (versus baseline)
**M0**	26	1284.80 [320.44-5373.14]	-	27	29.74 [16.24-38.03]	-	25	22.10 [16.68-30.02]	--
**M1**	24	177.38 [118.11-273.93]	0.004	27	25.45 [15.65-38.85]	0.231	27	19.56 [14.96-26.89]	0.214
**M6**	23	164.91 [110.34-213.39]	< 0.001	25	23.18 [16.66-41.99]	1.000	26	18.94 [15.31-29.23]	0.109

	**IL-1β**			**IL-3**			**IL-6**		
	n	Median [IQR]	*p*-value (versus baseline)	n	Median [IQR]	*p*-value (versus baseline)	n	Median [IQR]	*p*-value (versus baseline)
**M0**	25	7.75 [5.24-10.01]	-	27	1.83 [0.55-2.52]	-	24	6.40 [2.16-19.12]	-
**M1**	26	6.27 [2.21-8.80]	0.058	26	1.09 [0.59-2.41]	0.369	25	1.68 [1.13-2.75]	0.008
**M6**	25	6.87 [2.52-10.97]	0.087	21	0.92 [0.64-1.57]	0.428	25	1.28 [1.02-1.97]	0.002

		**IL-8**		**IL-18**			**TPX**		
	n	Median [IQR]	*p*-value (versus baseline)		Median [IQR]	*p*-value (versus baseline)	n	Median [IQR]	*p*-value (versus baseline)
**M0**	25	1.92 [0.82-2.81]	-	M0	304.34 [118.22-417.28]	-	26	756.42 [575.78-976.46]	-
**M1**	24	1.12 [0.59-1.36]	0.050	M1	132.78 [93.04-198.74]	< 0.001	27	541.36 [385.31-813.27]	0.055
**M6**	22	0.91 [0.66-1.48]	< 0.001	M6	114.88 [62.69-158.49]	< 0.001	27	460.98 [339.91-658.49]	< 0.001
